# Huge plastic bezoar: a rare cause of gastrointestinal obstruction

**DOI:** 10.11604/pamj.2015.21.286.7169

**Published:** 2015-08-18

**Authors:** Mbarek Yaka, Abdelkader Ehirchiou, Tariq Tajdin Sifeddine Alkandry, Khalid Sair

**Affiliations:** 1 Depatement of Surgery, Military Hospital Med, Rabat, Morocco

**Keywords:** Plastic bezoars, Huge abdominal lump, Bowel obstruction

## Abstract

Bezoars are rare causes of gastrointestinal obstruction. Basically, they are of four types: trichobezoars, phytobezoars, pharmacobezoars, and lactobezoars. Some rare types of bezoars are also known. In this article a unique case of plastic bezoars is presented. We describe a girl aged 14 years who ingested large amounts of plastic material used for knitting chairs and charpoys. The conglomerate of plastic threads, entrapped food material and other debris, formed a huge mass occupying the whole stomach and extended into small bowel.

## Introduction

Bezoars are rare causes of gastrointestinal obstruction. They mostly originate in the stomach, and occur mainly in patients with psychiatric ailments who chew and swallow their hair (trichobezoar), vegetable fibres (phytobezoar), persimmon fibres (diospyrobezoar), or tablets/semi liquid masses of drugs (pharmacobezoar). Industrial materials including wood trashes, polystyrene, plastic have been reported as the rare causes of bezoar formation. In this report, we describe a 14-year-old girl who developed obstructing Plastic bezoars of both the stomach and small intestine and literature review.

## Patient and observation

A 14-year-old girl presented with upper abdominal pain and vomiting for 15 days and absolute constipation for 7 days. Examination revealed pallor, tachycardia and a 20 cm firm, smooth, non-tender lump palpable in the epigastrium, moving with respiration. An ultrasound of the abdomen confirmed the presence of a large intragastric mass and an abdominal computed tomography (CT) scan revealed a heterogeneous gastric mass.

**Figure 1:**
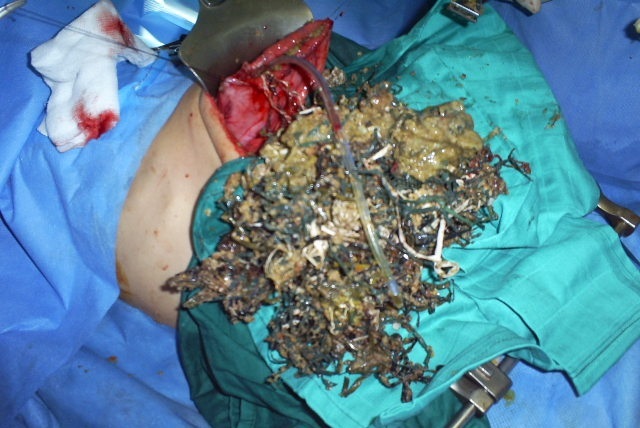
Large bezoar removed from stomach

**Figure 2:**
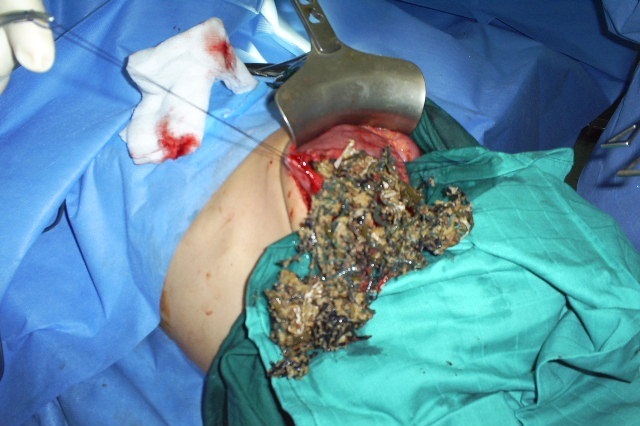
Photograph shows the Plastic bezoar visible through the gastrotomy

**Figure 3:**
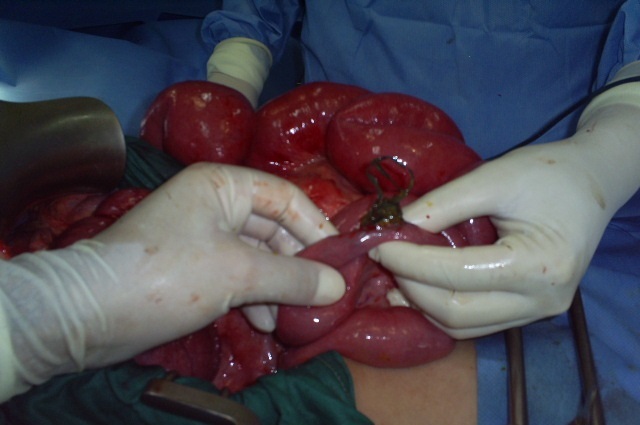
Plastic bezoar removed from small intestine

**Figure 4:**
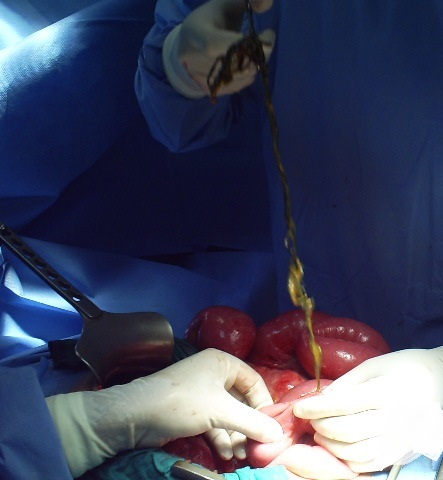
The tail of the bezoar pulled back from the intestine

At laparotomy, a bezoar made up of plastic material extended throughout the length of the stomach (Figure 1) and small bowel with two perforations of jejunum. The bezoar was removed enmasse through gastrotomy (Figure 2) with assisted multiple enterotomies (Figure 3, Figure 4). The segment of small bowel bearing perforations was resected and primary end-to-end anastomosis was done. After a thorough peritoneal lavage the abdomen was closed with drains in situ. The postoperative period was uneventful and patent was discharged in four days after surgery. She also underwent regular psychiatric counseling and s well one year after surgery. On inquiring from the parents, the Mather said that she had occasionally seen the girl peeling off plastic material from chairs and charpoys and eating it for the last two years.

## Discussion

The word bezoar is a word derived from the Arabic “bazahr” or “badzehr” which means an antidote or counter-poison. Till the 19^th^ century, bezoars obtained from sacrificed animals had been widely used as antidote [1].

Bezoars are the uncommon result of ingestion of poorly digestible or indigestible substances. Trichobezoars and phytobezoars are the two most common bezoars described in literature. Other unusual bezoars are pharmacobezoars, lactobe- zoars, metal bezoars, plastic bezoars, and sand bezoars. Trichobezoars are found in teenagers, especially in female patients with psychiatric problems. Phytobezoars are found more frequently and are incarcerated foodstuffs not digested in the intestine [1, 2]. Plastic bezoars are also rare, resulting from the ingestion of plastic material, especially by mentally retarded patients. The case in discussion had swallowed a large amount of plastic material and since plastic cannot be digested, a bezoar formed in the stomach, and the long strings extend down to the duodenum and intestines, leading to Rapunzel syndrome [3].

Predisposing factors to bezoars, in addition to dietary behaviour, include previous gastric surgery, particularly partial gastrectomy or truncal vagotomy with pyloroplasty. In adults, bezoars are most frequently encountered after gastric operations. In children they are associated with pica, mental retardation, psychiatric disorder and coeliac disease [3, 4]. Bezoars are usually found in the stomach but may also be found in the duodenum, ileum, jejunum, colon or Meckel′s diverticulum. They can be extremely large, cause a wide variety of symptoms and can be fatal. Patients with bezoars may present with gastrointestinal obstruction that may involve any part of the bowel. In childhood, undiagnosed gastric bezoars may result in serious complications. The history of foreign body ingestion, especially in children and mentally impaired patients, is important. Our patient had a unique bezoar due to eating disorder in which he was eating plastic material used for knitting chairs and charpoys. This abnormal eating disorder of plastikophagia has been reported earlier too [5].

Patients usually present with recurrent vomiting, abdominal pain, and sometimes lump in the abdomen. The diagnosis often can be made on the basis of findings of conventional radiography and barium studies. On plain abdominal radiography, we found an opaque bezoar, which formed a perfect cast of the stomach. CT-scan could demonstrate a well-defined round mass which could be outlined by stomach or the bowel wall and present characteristic internal gas bubbles of bezoars [6].

Endoscopic investigations could show all of gastric bezoars. Bezoars located in the esophagus or stomach should be treated conservatively in the first instance. Surgery is recommended in cases with massive and non-progressive foreign bodies, or complicated cases presenting with perforation, penetration, hemorrhage, or obstruction [3, 7].

## Conclusion

Plastic bezoars are rare causes of gastrointestinal obstruction, resulting from the ingestion of plastic material, especially by mentally retarded patients. Surgery is recommended in cases with massive and non-progressive foreign bodies, or complicated cases.
